# The value of NIPT combined with serum cell-free DNA, estriol, AFP, and b-HCG levels in the recognition of trisomy 21 and 18 in the second trimester

**DOI:** 10.5937/jomb0-33027

**Published:** 2023-10-27

**Authors:** JingLi Fu, XiaoYan Zhong, Dan Li, YunSheng Ge, XueQin Zhang

**Affiliations:** 1 Xiamen University, School of Medicine, Women and Children's Hospital, Department of Obstetrics, Xiamen City, China; 2 Xiamen University, School of Medicine, Women and Children's Hospital, Department of Central Laboratory, Xiamen City, China

**Keywords:** noninvasive prenatal testing from DNA (NIPT), serum screening, trisomy syndrome, neinvazivno prenatalno testiranje iz DNK (NIPT), skrining seruma, sindrom trizomije

## Abstract

**Background:**

This study aimed to evaluate the clinical application value of noninvasive prenatal testing from DNA (NIPT) and serum screening for screening in detecting fetal trisomy 21 and 18.

**Methods:**

As a retrospective analysis, we collected data from 1383 women (singleton pregnancy) who underwent serum screening and noninvasive prenatal testing from DNA (NIPT) in our department from May 2015 to September 2017 and calculated the diagnostic value of the two methods.

## Abstract (sr)

**Uvod:**

Ova studija je imala za cilj da proceni vrednost kliničke primene neinvazivnog prenatalnog testiranja iz DNK (NIPT) i serumskog skrininga u detekciji fetalne trizomije 21 i 18.

**Metode:**

U okviru retrospektvine analize smo prikupili podatke od 1383 žene (jednoplodna trudno}a) koje su bile podvrgnute skriningu seruma i neinvazivnom prenatalnom testiranju iz DNK (NIPT) u na{em odeljenju od maja 2015. do septembra 2017. godine i izračunali dijagnostičku vrednost dve metode.

**Rezultati:**

U 1383 slučaja, osetljivost skrininga seruma na trizomiju 21 i 18 je bila 76,9%, specifičnost 74,7%, PPV 2,9%, NPV 99,7% i AUC 0,758 (95% CI: 0,625-0,891). Osetljivost neinvazivnog prenatalnog testiranja iz DNK (NIPT) na trizomiju 21 i 18 je bila 100%, specifičnost 99,8%, PPV 81%, NPV 100% i AUC 0,999 (95% CI: 0,000-1,000).

**Zaključak:**

Skrining seruma može na vreme da otkrije visokorizične trudnice, ali je učestalost lažno pozitivnih i negativnih rezultata velika; tačnost neinvazivnog prenatalnog testiranja iz DNK (NIPT) je visoka, što može efikasno da smanji stopu rađanja dece sa poremećajima.

## Introduction

Trisomy syndrome is one of the most common chromosomal disorders, mainly manifested in the number of individual chromosomes and structural abnormalities in children are not integers. After suffering from this disease, lethargy, and feeding difficulties, its mental retardation performance is gradually evident with age, while IQ 25∼50, motor development, and sexual development are delayed [1]. According to the number of chromosomes, it can be separated into 21, 18, 13-trisomy, etc. This analysis focuses on the most common 21 [2] and 18-trisomy. In China, the incidence of trisomy syndrome is 0.13%-0.16% [3], and about 40% of infants with trisomy are born. Congenital disabilities seriously affect children's survival and quality of life and place a substantial financial burden on families; therefore, scientific prenatal screening and diagnosis are of great significance. Current prenatal screening mainly includes serum screening [4] [5] and invasive and noninvasive screening methods.

Serum screening refers to serum collection from the peripheral blood of pregnant women to detect AFP, uE3, and β-HCG levels in serum, which is generally conducted in the second trimester. This method is straightforward, noninvasive, and economical and helps us screen high-risk pregnant women from a large group [6] [7]. However, false negatives of this method are likely to be ignored, which may cause the birth with congenital disabilities. Therefore, we conducted a retrospective analysis focusing on false negatives of serum screening.

The gold standard for diagnosing trisomy syndrome is an invasive prenatal diagnosis, mainly including amniocentesis [8] [9] and chorionic villus biopsy. Its advantage is detection with high accuracy. However, it is an invasive detection method that can lead to abortion and infection in rare cases [10]. This is also the main reason hindering its widespread promotion. So, we started looking for new detection methods. Since Lo YM [11] found cell-free fetal DNA (cffDNA) fragments in the peripheral blood of pregnant women in 1997, new detection methods have been developed rapidly. This method is now the NIPT [12], which extracts cffDNA fragments in the peripheral blood of pregnant women, and performs highthroughput sequencing on DNA to obtain genetic information, thereby detecting whether the fetus has chromosomal diseases. The most significant advantage of NIPT is the noninvasive methodology. In recent years, NIPT has become a hot spot in research of NIPT-positive cases that were recommended to undergo invasive prenatal diagnosis [13] [14]. But most of them focus on the diagnostic value of NIPT for trisomy 21, and only a tiny part studies trisomy 18 and 13. Studies have shown that when NIPT is used for trisomy 18 and sex chromosome aneuploidy, its accuracy will decrease. To verify whether it is the case, we conducted this analysis.

In this retrospective analysis, we objectively analyzed the value of serum screening and NIPT for testing trisomy 21 and 18 and provided evidence for seeking a more straightforward diagnostic method.

## Materials and methods

### General information

This study was designed as a retrospective analysis of 1503 single pregnant women who received prenatal screening in Xiamen Maternal and Child Health Hospital from May 2015 to September 2017. Inclusion criteria: received both serum screening and NIPT, set up files in Xiamen Maternal and Child Health Hospital and regular pregnancy checkup, pregnancy outcome is clear with complete clinical data. Exclusion criteria: multiple pregnancies, fetal chromosome aneuploidy, pregnant women receiving an allogeneic blood transfusion, transplantation, and cell therapy. According to NIPT results, they were separated into two groups. The NIPT-positive group was subjected to invasive prenatal diagnoses, such as chorionic villus sampling (CVS) or amniocentesis, and the NIPT-negative group was followed up until the pregnant woman gave birth. As it was a retrospective analysis, we obtained no written informed consent. Before analysis, all records were anonymized and deidentified.

### Serum screening

We established a schedule for pregnancy checkups for pregnant women and strictly followed it. At 13-16 weeks of pregnancy, we drew about 4 mL of pregnant women's venous blood. We did not anticoagulate the blood. We extracted serum after centrifugation which received unified testing by the experimental center of our hospital. We adopted a corresponding enzyme-linked immunosorbent assay (ELISA) kit to detect AFP, β-hCG, and uE3 levels in serum and carried out experimental procedures strictly following instructions. We used Lifecycle 3.2 for risk calculation to process data to obtain the median concentration of each indicator. We made multiple of median (MOM) for AFP>0.65, β-hCG>2.65, uE3<2.50 screening criteria. Risk cut-off values of 21 and 18-trisomy were 1:270 and 1:350, respectively.

### NIPT

Between the 13^th^ and 22^nd^ weeks of pregnancy, we collected approximately 10 mL of peripheral blood of pregnant women, placed it in a centrifuge tube containing EDTA, and centrifuged them at 16000×g for 10 minutes. We separated the supernatant and placed it into a new centrifuge tube (without EDTA) to continue centrifugation at 16000×g for 10 min to collect the supernatant. We took 2 mL of the supernatant for the next analysis and stored the remaining supernatant at -80°C. We adopted QlAamp Circulating Nucleic Acid Kit (Qiagen) to extract cffDNA from plasma of 2 mL. We used Qubit Fhiorometer to detect cffDNA concentration. We constructed a DNA library according to instructions from the manufacturer. We performed high-throughput sequencing on the DNA library to obtain the number of DNA fragments distributed on each chromosome, combined with biological information analysis, calculated trisomy risk index Z, and determined whether the fetus was at risk of trisomy syndrome. When Z was in the range of -3–3, it was judged as a low-risk sample. When Z>3, the sample was considered high-risk [15].

### Data analysis

We used SPSS 23.0 software package for statistical analysis. We presented measurement data as mean ± standard deviation (x̄±Sd) and recorded count data as rate (%). Two-sided P<0.05 had statistical significance.

## Results

### General clinical data of research objects

**Figure 1:**
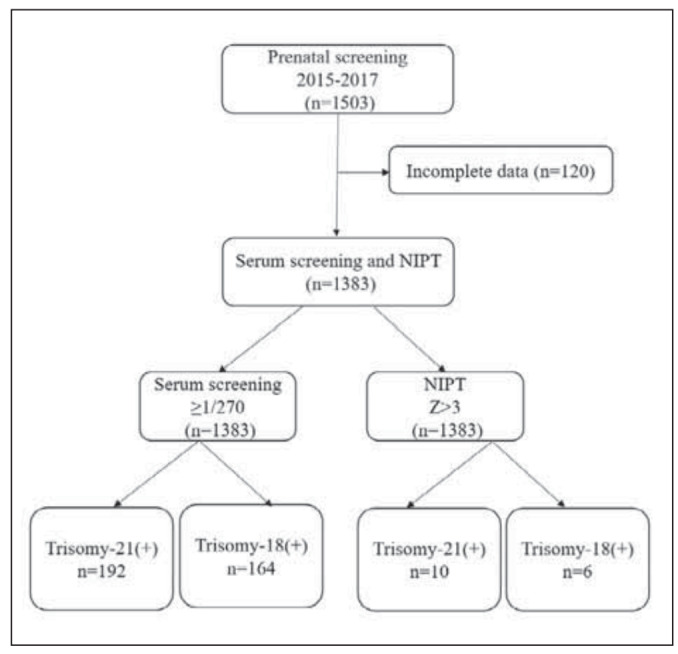
Combined diagram of the research process for prenatal screening of patients.

From May 2015 to September 2017, in total, 1503 pregnant women received prenatal screening in our hospital, of which 1383 received serum screening and NIPT. Data was valid and included in our analysis. The remaining 120 pregnant women who received only one of them were excluded. The median age of pregnant women was 31 years (20-49); 789 were younger than 35 years old, and 594 were 35 years old or older. Median age of fetus was 16 weeks (12–22). About 41.2% of pregnant women were admitted to our hospital during the first trimester, and the remaining 58.2% during the second. A comprehensive analysis of chromosome karyotype via amniocentesis and follow-up results after delivery suggested that 1369 fetuses were euploid and 14 were aneuploid. The cases of aneuploidy included 8 instances of 21-trisomy, 5 cases of 18-trisomy, and 1 case of 13-trisomy. The prevalence was 0.6%, 0.4%, and 0.1%, respectively (Table 1, Figure 1).

**Table 1:** Basic information of study objectives (n=1383). Note: The value is the median (range) or number (%).

classification	data
Age of pregnant women, yrs.	31 (20–49)
<35	789 (57.0)
≥35	594 (43.0)
Gestational age, wks.	16 (12–22)
First trimester	570 (41.2)
Second trimester	813 (58.2)
BMI, kg/m^2^	21.4 (18.1–45.6)
Chromosome karyotype	
normal	1369 98.9
Trisomy-21	8 0.6
Trisomy-18	5 0.4
Trisomy-13	1 0.1

### Incidence of trisomy-21 with different risk values

In actual clinical work, many pregnant women have doubts about risk value. As long as the risk value is high, they are worried about whether they will give birth to defective children. Therefore, we counted how many people were with different risk values for each age group and compared the probability of trisomy-21 and 18 with varying values of risk. We separated pregnant women into two groups based on their age, less than 35 years of age and 35 years of age or older. Following many studies, we separated the risk value of serum screening for trisomy-21 into three segments: risk cut-off value 1/270, 1/270–1/1000, and <1/1000. We selected the risk cut-off value 1/270 of trisomy-21 in this analysis and screened 192 high-risk pregnant women. Among them, 77 cases of pregnant women were younger than 35, and the incidence of trisomy-21 was 2.60%; 115 cases of pregnant women were older than 35, and the incidence of trisomy-21 was 3.48%. There were 340 pregnant women in the risk range of 1/270–1/1000, 123 pregnant women younger than 35, and no trisomy-21 was detected; 217 pregnant women were 35 or older, and one pregnant woman whose fetus was diagnosed as trisomy-21 was detected, with the incidence of 0.46%. The last risk interval was <1/1000, which was the safest interval where there were in total of 851 pregnant women, with 589 cases of pregnant women younger than 35 years old, and no trisomy-21 detected; 262 pregnant women 35 years of age or older, and one pregnant woman whose fetus was diagnosed as trisomy-21 detected, with the incidence of 0.38%. We found that trisomy-21 risk cut-off value 1/270 selected in this analysis missed two cases, and there was one pregnant woman whose fetus was diagnosed as trisomy-21 at risk ranges of 1/270-1/1000 and <1/1000 (Table 2).

**Table 2:** Comparison of trisomy-21 incidence with different risk values.

Groups	Case of serum<br>screening (n)		Diagnosed<br>case (n)		Incidence<br>(%)	
	<35	≥35	<35	≥35	<35	≥35
≥1/270	77	115	2	4	2.60	3.48
1/270–1/1000	123	217	0	1	0	0.46
<1/1000	589	262	0	1	0	0.38

### Incidence of trisomy-18 with different risk values

We separated serum screening for 18-trisomy risk value into three segments: risk cut-off value 1/350, 1/350–1/1000, and <1/1000 (Table 3). Using an 18-trisomy risk cut-off value of 1/350 could screen 164 high-risk pregnant women from 1383 pregnant women. We separated high-risk pregnant women into two groups according to their age, less than 35 years of age and 35 years of age or older. There were 97 high-risk pregnant women less than 35 years of age, with an incidence of trisomy-18 of 2.06%; there were 67 cases 35 years of age or older, with an incidence of trisomy-18 of 2.99%. In the range of 1/350–1/1000, there were 443 pregnant women, of which 154 were younger than 35, and 289 were 35 years of age or older. One fetus was diagnosed with trisomy-18, and the incidence was 0.35%. The last risk interval was <1/1000, and there were 776 pregnant women, 538 pregnant women less than 35 years old, 238 pregnant women were 35 years of age or older, and no trisomy-18 was detected (Table 3).

**Table 3:** Comparison of trisomy-18 incidence with different risk values.

Groups	Case of serum<br>screening (n)		Diagnosed<br>case (n)		Incidence<br>(%)	
	<35	≥35	<35	≥35	<35	≥35
≥1/270	77	115	2	4	2.60	3.48
1/270–1/1000	123	217	0	1	0	0.46
<1/1000	589	262	0	1	0	0.38

### NIPT results of 21 and 18-trisomy

All pregnant women received NIPT, and 16 pregnant women tested positive. Of those 16 pregnant women, 14 were high-risk in serum screening, and the remaining two were pregnant women with low-risk serum screening. We performed chromosome karyotype analysis via amniocentesis on pregnant women who tested positive via NIPT screening, and those who tested negative via NIPT screening were followed up by telephone until delivery. Among 16 high-risk pregnant women in NIPT, 10 were at high risk of trisomy 21. In the final karyotype analysis, 8 cases were diagnosed as trisomy 21, and the remaining two had normal fetal chromosomes. Six cases were at high risk of trisomy 18, but 5 patients were finally diagnosed with trisomy 18, and the remaining 1 had normal fetal chromosomes. The total number of positive cases in NIPT screening for 21 and 18-trisomy was 16, with 13 actual positive cases and 3 false positive cases (Table 4).

**Table 4:** NIPT results of 21 and 18-trisomy (case).

Abnormal<br>type	High-risk case<br>in NIPT	chromosome karyotype<br>analysis	
		abnormal	normal
Trisomy-21	10	8	2
Trisomy-18	6	5	1
**In total**	**16**	**13**	**3**

### Effectiveness evaluation of serum screening and NIPT in the diagnosis of trisomy 21 and 18

**Figure 2:**
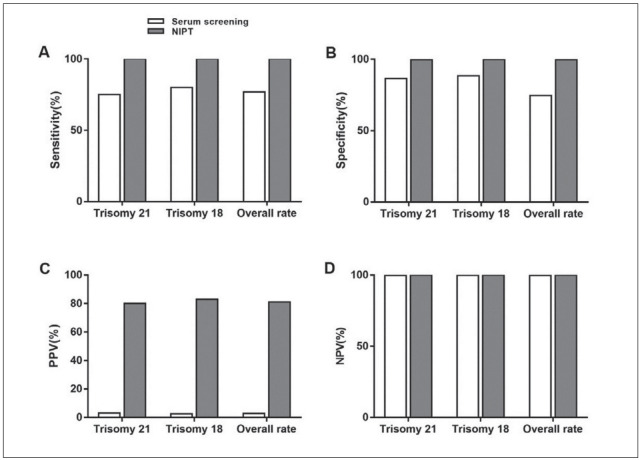
Function of serum screening and NIPT in the detection of trisomy 21 and 18: Statistics of sensitivity (A), specificity (B), PPV (positive predictive value, C) as well as NPV (negative predictive value, D) of serum screening and NIPT to trisomy 21 and 18, and the sum of the two.

**Figure 3:**
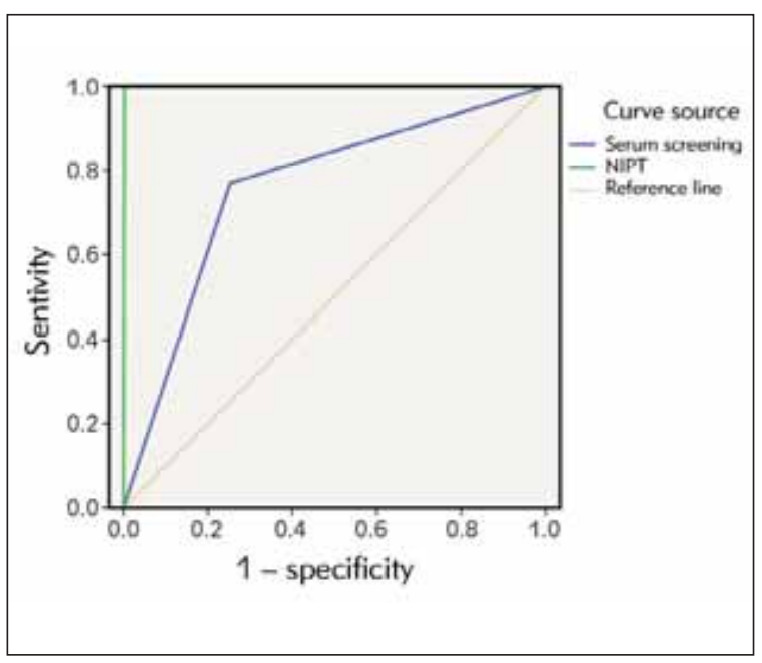
ROC curve of diagnostic efficacy of serum screening and NIPT for trisomy 21 and 18

The sensitivity of NIPT to trisomy 21 and 18 was 100% (13/13), specificity 99.8% (1367/1370), PPV 81% (13/16), NPV 100% (1367/1367). The sensitivity of serum screening to trisomy 21 and 18 was 76.9% (10/13), specificity 74.7% (1024/1370), PPV 2.9% (10/349), and NPV 99.7% (1024/1027), there were indeed two false negative cases. We constructed a ROC curve and compared the value of two diagnostic methods to identify trisomy 21 and 18. The AUC value of serum screening was 0.758 (95% CI: 0.625-0.891), and that of NIPT was 0.999 (95% CI: 0.000-1.000) (Figure 2, Figure 3).

### Pregnancy outcomes of research objectives

In our research groups, 2 cases (0.14%) caused fetal death due to spontaneous abortion or stillbirth in the second or third trimester. For patients where results of karyotype analysis via amniocentesis presented aneuploidy, we recommended termination of pregnancy. In the end, we chose 10 cases (0.72%) to terminate the pregnancy, but 2 cases persisted in continuing the pregnancy, who eventually all gave birth to defective babies, with one affected by 21-trisomy and the other 18-trisomy. The remaining 1396 pregnant women gave birth normally and g to fetuses with euploid (Table 5).

**Table 5:** Pregnancy outcomes of research objectives (n=1383).

Outcomes	Cases (%)
Normal pregnancy	1371(99.1)
Spontaneous abortion	1(0.1)
Dead fetuses	1(0.1)
Termination of pregnancy	10(0.7)
Natural labor	797(58.1)
Cesarean delivery	574(41.9)

## Discussion

We demonstrated how clinically applied serum screening and NIPT was used to detect whether fetuses were infected by trisomy 21 and 18 in the first or second trimester of pregnancy. We adopted outcomes of fetal karyotype and pregnancy to evaluate the sensitivity and specificity of the two methods. Our findings presented that the sensitivity of serum screening to trisomy 21 and 18 was 75% and 80%, respectively, and specificity was 86.5% and 88.4%, respectively, which were similar to those of other population-based studies using serum screening. In serum screening for trisomy 21 and 18, false positives and negatives were too high. Studies have shown that false positives in serum screening could affect the mood of pregnant women [16]. In this analysis, we screened for 356 high-risk ones from 1383 pregnant women, the sum of 192 cases of high-risk T21 and 164 high-risk T18 in serum screening, which affected most pregnant women both physically and mentally. They were terrified, and it was not conducive to pregnancy. For high-risk pregnant women, clinicians should actively talk to pregnant women not to worry about it overly. Among analyzed cases, two tested false-negative, which fortunately was detected via NIPT and thus avoided the birth of a defective child. In recent years, some pregnant women finally gave birth to children with trisomy syndrome due to false negatives detected through serum screening. It reminds us to pay attention to false negatives in serum screening. For low-risk pregnant women in serum screening, we should take it seriously. To avoid the occurrence of false negatives, it is necessary to strengthen the quality control of all aspects of prenatal, combined with NIPT and other tests and the comprehensive assessment of maternal history, to minimize the birth rate of Down's children and reduce the burden on pregnant women's families.

Next, we probed into why false positives and negatives were high in serum screening were so high in number. There were three main serum screening indicators: AFP, uE3, and β-HCG. We used ELISA for detection with high sensitivity, which is a possible factor for high false positives in serum screening. In addition, AFP is a special protein that can prevent a fetus from being rejected by the mother. In the first trimester, AFP is only expressed in the yolk sac. At 6w, the liver of the fetus is capable of gradually expressing AFP until it reaches a peak at 13w. In pregnant women with children infected by trisomy syndrome, the AFP level in blood is 0.7 to 0.8 times that of normal pregnant women. AFP in the peripheral blood of pregnant women mainly comes from amniotic fluid [17], and it in amniotic fluid comes from fetal urine; that is, AFP level in amniotic fluid is approximately equal to that in fetal blood. However, AFP in amniotic fluid and maternal blood is not exactly the same. As a human chorionic gonadotropin synthesized by placental cells [18] [19], hGG is a key marker reflecting fertilized egg implantation and has two subunits: and β. We tended to detect β subunit, which could reflect the state of the placenta and fetus. uE3 [20] is synthesized by the fetal-placental unit of the fetus. As pregnancy progresses, placental function gradually develops, and the uE3 level also jumps. Thereby, the uE3 level can monitor the fetus's growth status.

Pregnant women with children infected by trisomy syndrome have 30% lower blood AFP levels than normal pregnant women. It now appears that these three indicators could represent some feedback about the fetus in the placenta. However, this relative value will become inaccurate due to individual differences and complicated maternal-fetal physiology. That is why the sensitivity and specificity of serum screening are not high. There is no doubt that serum screening is very valuable in reference, but we still need to combine it with other prenatal diagnostic methods, such as NIPT, to make its best use.

Therefore, our retrospective analysis also analyzed NIPT value in detecting trisomy 21 and 18. We adopted NIPT through cffDNA in the peripheral blood of pregnant women to detect trisomy 21 and 18. Findings presented that detection sensitivity was 100% and 100%, and specificity was 99.8% and 99.9%, respectively. Based on a meta-analysis, the detection rate of trisomy 21 was 99.5%, and that of trisomy 18 was 97.7% [21] [22]. The sensitivity and specificity of NIPT detection for trisomy 21 and 18 were very high, and there were still low false positives. The reasons might be as follows: At 5w, there was a small amount of cffDNA in the peripheral blood of pregnant women. Derived from villi cells, cffDNA concentration increases with increasing gestational age [23] and can exist in the maternal blood until one day after delivery; it is cleared quickly via the mother's immune system. The proportion of fetal components in cffDNA is 13.7%, and some DNA fragments are derived from the placenta [24]. Some pregnant women also engage in physical labor or have pregnancy complications, which could lead to placentalderived DNA to account for as high as 35% [25], affecting NIPT accuracy.

This analysis is mainly to evaluate the value of serum screening and NIPT. As for the disadvantage, we did not analyze the combined value of the two. Various studies have proved that combining serum screening and NIPT could improve detection accuracy. Nevertheless, the more inspection items, the increase in medical costs, which is not conducive to promotion.

In summary, serum screening and NIPT could effectively detect trisomy 21 and 18, and contribute to reducing the birth rate of defective fetuses, but we still need to improve them for promotion continuously.

## Dodatak

### Ethics statement

The experiment was approved by the Ethics Committee of Xiamen Maternal and Child Health Hospital in December 2014, and all patients participating in this study provided written informed consent following the »Helsinki Declaration.« The approval number is 201412022HL.

### Conflict of interest statement

All the authors declare that they have no conflictof interest in this work.
